# Ozone adjunct treatment in facing multidrug‐resistant bacteria?

**DOI:** 10.1002/ccr3.8087

**Published:** 2023-10-18

**Authors:** Salvatore Chirumbolo, Luigi Valdenassi, Marianno Franzini

**Affiliations:** ^1^ Department of Engineering for Innovation Medicine University of Verona Verona Italy; ^2^ Italian Scientific Society of Oxygen‐Ozone Therapy (SIOOT) Bergamo Italy

To the Editor,

A recent paper on this journal reported a successful anti‐septic treatment on a case of necrotizing fasciitis caused by *Streptococcus pyogenes* infection in an 88‐year‐old woman.^1^ The patient suffered an *S. faecalis* infected scar for 15 days on the left tibia, making her unable to stand. The leg appeared inflamed and painful, with a 3 cm wound exhibiting a necrotic area on the left heel.

Antibiotic treatment consisted in amoxicillin 8 g/day on hospital Day 1 (without clavulanic acid) and clindamycin 1.2 g/day for 15 days.^1^ Following this treatment, the patient did not fully ameliorate her wound aspect and stenosis, yet the wound was washed, treated with alginate‐based dressings and fibrin removed, then some improvement could be observed. The antibiotic treatment failed in reducing the bacterial inflammation. Increasing data about antibiotic resistance to clindamycin in group A streptococci should raise some concerning comment on this report.^2^

Ozone, an allotrope of oxygen, is heightening its notoriety as a chemical compound able to successfully address the great concern of multidrug‐resistant bacteria (MRB) for public health.^3^ The use of ozone as an ambient disinfectant is only one of the numerous possibilities to include this chemical in the treatment of MRB, particularly in hospital care. Medical ozone should be distinguished from chemical ozone used as an ambient disinfectant, as it is employed as an adjunct therapy in cleansing and removing septic bacteria from wounds, particularly post‐surgical wounds.^4^, ^5^

Some evidence has been reported showing that low doses of an oxygen–ozone mixture are able to eradicate many fundamental pathological bacteria, such as *Escherichia coli*, carbapenem‐resistant *Acinetobacter baumannii*, vancomycin‐resistant *Enterococcus faecalis*, *Klebsiella pneumonia, Pseudomonas aeruginosa* susceptible to meropenem and imipenem, and so forth.^6^ Low doses of oxygen–ozone represent the medical approach with which a balanced oxygen–ozone mixture injected in tissues, including peripheral blood, elicit, via the ozone‐generated 4‐hydroxynonenal (4‐HNE), a hormetic response, leading cells to mechanisms of survival including the promotion of mitophagy, the modulation of inflammation by the inflammasome NLRP3, and the inhibition of pyroptosis.^7^

While ozone, at relatively high doses, is enabled to kill bacteria from surfaces, its action on mitochondria of innate immune cells and endothelia, once oxygen–ozone is used as an adjunct therapy against bacterial infections, is paramount, because ozone is able to modulate, by 4‐HNE, the ability of cells to respond to oxidative stress and inflammation, therefore contributing in dampening any painful inflammatory response. The high pleiotropism and unspecific action against bacteria may put ozone in the spotlight as a possible candidate in addressing MRB concern.^8^

A correct and successful innate immune response against bacteria needs a proper mitochondria activity, particularly regarding mitochondria autophagy or mitophagy.^9^

A delicate balance between apoptosis (death) and survival is finely regulated by mitochondria, via the mitophagy function, even in the cardiovascular system, where mitophagy acts as a cardioprotective system, whereas an excessive oxidative stress destroys this balance.^10^ Defects in the mitophagy regulation impair also the ability of innate immunity to address bacterial infections, via the PINK1/Parkin pathway.^11^ Mitochondria are the central hub to regulate innate immunity and immune tolerance, the latter in case of mitochondria functional impairment.^12^

Noteworthy, MRB may be settled by a concurrent interplay of mitochondria dysfunction, mitophagy impairment, and excess of oxidative stress, causing a situation of immune tolerance, which exacerbates the bacterial colonization and inflammatory response.

In this micro‐environment, low and hormetic doses of ozone may tune the correct activity of mitochondria on the redoxin‐mediated process of oxidative stress response, so generating a pro‐survival and anti‐inflammatory response, adjusting mitophagy by 4‐HNE.

Necrotizing fasciitis can be even treated with ozone, as previously demonstrated.^13^

Considering ozone as a bioregulator is an exciting possibility to address MRB therapy and remove bacterial infection from post‐surgical wounds, by using oxygen–ozone as an adjunct therapy.

To date, several interesting technologies regarding the use of ozone against bacteria are available. For example, wearable, portable topical ozone devices, developed at Purdue University, are available for the treatment of non‐healing and infected wounds, as a patch system.^14^ Nanoparticles able to make ozone available against MRD bacteria are another possibility to use in a reliable and highly efficient way ozone against sepsis‐causing microbial agents.

As MRB is a huge concern for public health and the global healthcare policy, science should try new enterprises and suggestions to overcome this burdensome issue. Ozone appears as a promising candidate, both for its direct ability in killing bacteria and its potential in regulating patients' immune response, drastically reducing the progress toward a painful exacerbation of the bacterial infection.

## AUTHOR CONTRIBUTIONS

**Salvatore Chirumbolo:** Conceptualization; data curation; investigation; methodology; supervision; validation; visualization; writing – original draft; writing – review and editing. **Luigi Valdenassi:** Investigation; supervision; validation; visualization. **Marianno Franzini:** Formal analysis; investigation; project administration; software; supervision; visualization.

## Data Availability

Not applicable.
